# Calcium Chloride as a Novel Stabilizer for Foot-and-Mouth Disease Virus and Its Application in the Vaccine Formulation

**DOI:** 10.3390/vaccines12040367

**Published:** 2024-03-29

**Authors:** Jong Sook Jin, Gyeongmin Lee, Jae Young Kim, SooAh Lee, Jong-Hyeon Park, Sun Young Park, Young-Joon Ko

**Affiliations:** Center for FMD Vaccine Research, Animal and Plant Quarantine Agency, 177 Hyeoksin-8-ro, Gimcheon-si 39660, Republic of Korea; in75724@korea.kr (J.S.J.); lgm6004@korea.kr (G.L.); ivorikim@korea.kr (J.Y.K.); 0902lsa@korea.kr (S.L.); parkjhvet@korea.kr (J.-H.P.)

**Keywords:** foot-and-mouth disease virus, vaccine, storage, stabilizer, calcium chloride

## Abstract

The thermal stability of the in-house-developed foot-and-mouth disease (FMD) type O and A viruses was evaluated, and the O Jincheon virus was found to exhibit the lowest thermal stability. To overcome this instability, we proposed a novel stabilizer, calcium chloride. The thermal stability of FMDVs increased up to a CaCl_2_ concentration of 10 mM, and it had a decreasing trend at >30 mM. The O Jincheon virus showed a significant decrease in the amount of antigen over time at 4 °C. In contrast, the samples treated with CaCl_2_ showed stable preservation of the virus without significant antigen loss. After the CaCl_2_-formulated vaccine was administered twice to pigs, the virus neutralization titer reached approximately 1:1000, suggesting that the vaccine could protect pigs against the FMDV challenge. In summary, the O Jincheon virus is difficult to utilize as a vaccine given its low stability during storage after antigen production. However, following its treatment with CaCl_2_, it can be easily utilized as a vaccine. This study evaluated CaCl_2_ as a novel stabilizer in FMD vaccines and may contribute to the development of stable vaccine formulations, especially for inherently unstable FMDV strains.

## 1. Introduction

Foot-and-mouth disease (FMD) is a highly contagious viral disease that affects cloven-hoofed animals and poses a significant threat to the livestock industry worldwide. The causative agent, the FMD virus (FMDV), a member of the *Picornaviridae* family, possesses a single-stranded positive-sense RNA of approximately 8500 nucleotides [1]. FMDVs are known to have seven immunologically distinct serotypes (O, A, C, Asia1, SAT1, SAT2, and SAT3) and various subtypes owing to its genetic diversity [2]. The FMDV RNA genome contains four structural proteins (P1, VP4, VP2, VP3, and VP1) and eight non-structural proteins (L, 2A, 2 B, 2C, 3A, 3 B, 3C, and 3D) [3]. The VP1, VP2, and VP3 proteins contribute to the formation of the viral surface to present various antigenic epitopes, whereas the VP4 protein internally stabilizes the viral RNA within the capsid [4]. Each structural protein (SP) is linked to another to form a protomer (12S), five of which are assembled into pentamers (75S), and 12 pentamers form empty capsids (75S) and virions (146S) [5,6].

The development of effective FMD vaccines is crucial to controlling the spread of the virus and many countries have implemented FMD vaccination policies to control the widespread outbreaks, minimize economic losses, and ensure international trade in animals [7,8,9]. However, the physical properties of FMDVs, which are highly sensitive to environmental conditions, such as temperature and pH, may pose difficulties in developing an FMD vaccine. In particular, the FMDV 146S particle is compromised under mildly acidic conditions or elevated temperatures, leading to its easy dissociation into pentameric subunits [10,11,12], and the histidine residues at the pentamer-pentamer interface play a key role in this process [13,14]. Importantly, the dissociated 12S subunit has been reported to be less immunogenic and elicit a reduced neutralizing response [15,16]. This physical vulnerability of FMDVs ultimately leads to reduced vaccine efficacy and a shortened storage period; therefore, it is necessary to develop additives that improve FMDV stability.

Stabilizing additives such as disaccharides [17,18,19], amino acids [20], detergent [21], metal ions [19,22], etc. have been reported for various viruses, including FMDV, and research on stabilizing FMDVs by amino acid substitution of viral capsid proteins is being conducted worldwide [23,24,25]. Among these, divalent transition metal ions have been found to contribute to the stabilization of FMDV particles by forming salt bridges at the inter-pentameric interface of the FMDV capsid [26], which is known to vary depending on factors such as the virus strain [27]. Stabilization of the poliovirus by divalent cations has been demonstrated [28,29,30,31].

These previous studies showed some improvement in viral stability, but it has not been evaluated whether the vaccine antigen content is maintained after long-term storage in the aqueous phase and whether the excipients affect the immunogenicity of the vaccine.

Among the seven serotypes of FMDVs, type O is the predominant serotype worldwide, and outbreaks associated with the Southeast Asia (SEA/Mya-98 lineage), Middle East–South Asia (ME-SA/Pan-Asia and Ind 2001 lineage), and Cathay topotypes have been reported in Asia [32,33]. In particular, type O has been reported to have relatively lower stability than other FMDV serotypes [11,34]. Likewise, the obvious instability of the type O Jincheon virus (SEA/Mya-98 lineage), a Korean isolate, was confirmed to be highly labile in our previous study [35]. To overcome this, we developed a stabilizing agent—SLA, which is a mixture of sucrose and lactalbumin hydrolysate (SLA) [35]. However, since this is a composition that can support the growth of microorganisms, it was found to be vulnerable to the growth of bacteria and fungi, even when stored at 4 °C, and it forms protein precipitates. Therefore, we explored new stabilizers to replace SLA and found that calcium improved the physical stability of the O Jincheon virus.

This study investigated the inherent thermal stability of FMDVs, focusing on strains isolated in South Korea, to improve the stability of unstable FMDV strains using calcium chloride as a stabilizer. In addition, we evaluated the applicability of calcium chloride as an additive in FMD vaccines.

## 2. Materials and Methods

### 2.1. Cells and Reagent

Baby hamster kidney-21 (BHK-21) suspension cells were established in a serum-free medium from the original adherent BHK-21 cell line (ATCC, Manassas, VA, USA) by the Animal and Plant Quarantine Agency (APQA; Gimcheon-si, Republic of Korea) and the Korea Research Institute of Bioscience and Biotechnology (Chungju-si, Republic of Korea). The suspended cells were grown in CellVento medium (Merck, Darmstadt, Germany) and cultivated in a shaking incubator at 37 °C with 5% CO_2_. Calcium chloride was purchased from Sigma-Aldrich (St. Louis, MO, USA), dissolved in distilled water, and used after preparing a 300 mM stock.

### 2.2. Viruses

In this study, eight FMDV strains were used: five Korean isolates, O/SKR/Jincheon/2014 (GenBank accession no. 162590.1), O/SKR/Andong/2010 (GenBank accession no. KF112887.1), O/SKR/Boeun/2017 (GenBank accession no. MG983730.1), A/SKR/Yeoncheon/2017 (GenBank accession no. KY766148.1), and A/SKR/Pocheon/2010 (GenBank accession no. KC588943.1)), and three genetically engineered foreign-origin strains obtained by replacing the P1 region of O1/Manisa/Turkey/69 (GenBank accession no. AY593823), O PanAsia-2 (GenBank accession no. GU384682 for P1), O/Taiwan/97 (GenBank accession no. AY593823 for P1), and A_22_/Iraq/24/64 (GenBank accession no. AY593764.1 for P1), as previously reported [36,37]. BHK-21 suspension cells were cultured in CellVento medium starting with 3 × 10^5^ cells/mL until they reached 3 × 10^6^ cells/mL for 3.5 days and were inoculated with FMDVs with 30% (*v*/*v*) fresh CellVento medium. Viruses were harvested 16–20 h post-infection and clarified by centrifugation at 3000× *g* for 20 min to remove cell debris. FMDVs were inactivated by treatment with binary ethylenimine (BEI, Sigma-Aldrich) to a final concentration of 3 mM, and BEI was neutralized by adding 2% (*v*/*v*) sodium thiosulfate (Daejung Chemicals & Metals, Siheung, Republic of Korea).

### 2.3. Purification of FMDV 146S Particles

A clarified FMDV sample was treated with 7.5% polyethylene glycol 6000 (Sigma-Aldrich) to concentrate the virus particles [38]. The concentrated viral antigen was placed in a tube with a sucrose density gradient (SDG) of 15–45% and then ultracentrifuged at 100,000× *g* for 4 h at 4 °C. Centrifuged samples were fractionated using a continuous density gradient fractionator (Teledyne ISCO, Lincoln, Nebraska). The absorbance of each fraction was measured at 254 nm using a spectrophotometer component. At this time, the amount of 146S, a viral particle (μg/mL), was calculated by measuring the area under the peak of a specific fraction according to a previous study [39]. The peak fraction containing 146S particles was then collected and ultracentrifuged at 100,000× *g* for 4 h at 4 °C. The harvested virus pellet was resuspended in Tris-KCl buffer (pH 7.6).

### 2.4. Quantification of FMD Vaccine Antigen

FMDV vaccine antigens were quantified by size-exclusion high-performance liquid chromatography (SE-HPLC) on FMDV samples purified by ultracentrifugation as previously described [40]. Briefly, the gel column used in this study was a TSKgel G4000PWXL (300 mm × 7.8 mm I.D.) column (TOSOH Bioscience, Tokyo, Japan) equipped with a TSKgel PWXL Guardcol (40 mm × 6.0 mm) guard column (TOSOH Bioscience). Absorbance at 254 nm was applied using an Agilent 1260 Infinity II system (Agilent Technologies, Santa Clara, CA, USA). The mobile phase consists of 30 mM Tris-HCl and 400 mM NaCl (pH 8.0) at a flow rate of 0.5 mL/min. FMDV 146S particles were quantified by calculating the area under the target peak as previously described [41].

### 2.5. Thermal Stability Assay for FMDV

A particle stability thermal release assay (PaSTRy) was performed in 96-well plates using a CFX96 real-time PCR system (Bio-Rad, Hercules, CA, USA). SYBR green II dye, which binds to RNA, was used to detect the exposed RNA genome after capsid dissociation by increasing the temperature of the virus. Thereafter, 1 μg of purified FMDVs and 5 μL of 100× SYBR green II dye (Thermofisher Scientific, Dartford, UK) were mixed, and the volume was made up to 50 μL with 1× PBS (Corning, NY, USA). In experiments to determine the effect of calcium, an additional 300 mM of calcium chloride was added to dilute to final concentrations of 3, 10, 30, and 50 mM. The assay temperature was ramped up from 15 °C to 95 °C in 0.5 °C increments with intervals of 10 s, and fluorescence was read with excitation and emission wavelengths of 490 nm and 516 nm, respectively.

### 2.6. Preparation of FMD Vaccines

To prepare the FMD vaccine antigen, two additives based on Tris-KCl buffer were added to the purified FMDV O Jincheon virus: (i) 3 mM calcium chloride and (ii) 10% sucrose with 5% lactalbumin hydrolysate (SLA). The vaccine antigen was mixed with 1% saponin (Sigma-Aldrich) and 10% aluminum hydroxide gel (General Chemical, Moorestown, NJ, USA), and then, ISA 206 VG adjuvant (Seppic, Paris, France) was added in a 1:1 ratio to make 2 mL/dose of the test vaccine. After mixing, it was incubated at 20 °C for 1 h in a water bath without light exposure and then stored at 4 °C until use.

### 2.7. Immunization of Pigs

The efficacy of the FMD vaccine is determined by the viral particle called 146S. It is already known that if the intact 146S of FMDVs is degraded into the pentameric 12S, the efficacy of the FMD vaccine is significantly reduced [15]. Therefore, it was not necessary to evaluate the immunogenicity of samples with and without 12S obtained from the 4 °C storage experiment in pigs. In this study, we aimed to evaluate whether the addition of the calcium that increased the stability of the type O Jincheon virus could affect its immunogenicity in pigs. In addition, since the experimental vaccine was prepared by purifying and quantifying only 146S particles and inoculated into pigs at 15 ug/dose, the degraded form, 12S, was not included in the experimental vaccine.

Eighteen 8-week-old pigs confirmed to be negative for SP antibodies by ELISA were administered FMD vaccines containing calcium chloride (*n* = 6) or SLA (*n* = 6) by intramuscular injection. All pigs were vaccinated 2 times at a dose of 2 mL containing 15 μg of 146S antigens and bled at every week until 56 days after the initial vaccination.

### 2.8. Enzyme-Linked Immunosorbent Assay

The antibody levels induced by the FMD vaccines were measured using a PrioCHECK FMDV type O antibody detection kit (Prionics, Lelystad, The Netherlands), and competition enzyme-linked immunosorbent assay (ELISA) was conducted following the manufacturer’s instructions. Test sera were added to antigen-coated plates and incubated for 1 h at 25 °C. After the plates were washed, horseradish peroxidase-conjugated monoclonal antibodies against the immobilized type O FMDV SP antigen were added. The optical density was measured according to the colorimetric reaction at 450 nm.

### 2.9. Virus Neutralization Test

The procedure for testing FMDV antibodies using the virus neutralization test adhered to the guidelines outlined in the World Organization for Animal Health (WOAH) Terrestrial Manual [42]. Initially, serum samples were inactivated at 56 °C for 30 min before testing. Starting from a 1/8 dilution, sera are diluted in a twofold, dilution series across the plate, using two rows of wells per serum and a volume of 50 μL. These diluted samples were then mixed with an equal volume of FMDVs containing 100 TCID_50_ (50% tissue culture infective dose). After incubating at 37 °C for 1 h, porcine kidney cells (LFBK, supplied by the Plum Island Animal Disease Center (Orient, NY, USA)), at a concentration of 0.5 × 10^6^ cells/mL, were added into each well. The plates were sealed and incubated at 37 °C with 5% CO_2_ for 2–3 days. The virus neutralizing (VN) titer was calculated as the reciprocal of the maximum dilution of serum that neutralized 100 TCID_50_ of FMDV and was expressed as a log_10_ value using the Spearman–Karber method [43].

### 2.10. Statistical Analysis

Statistical data were analyzed using GraphPad Prism version 9 (GraphPad Software, La Jolla, CA, USA) for visual representation. Statistical significance was defined using an unpaired *t* test.

### 2.11. Ethic Statement

Animal experiments in this study were approved by the Institutional Animal Care and Use Committee of the Animal and Plant Quarantine Agency (IACUC) and performed in accordance with the National Institutes of Health Guide for the Care and Use of Laboratory Animals (IACUC approval no. 2023-677).

## 3. Results

### 3.1. Evaluation of FMDV Thermal Stability

**Figure 1:**
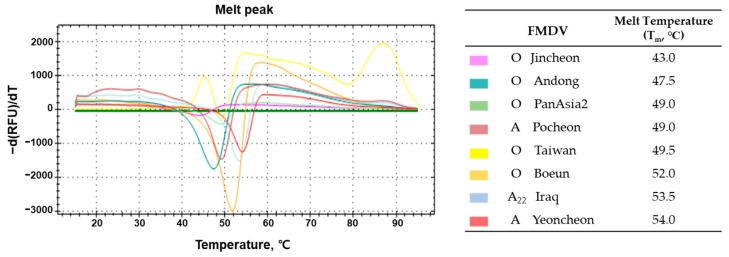
Analysis of thermal stability of FMD vaccine antigens. The thermal stability of eight strains of FMDVs was monitored by particle stability thermal release assay (PaSTRy). The viral RNA genome released from the capsid was detected by SYBR green II dye. The first derivative of fluorescence was plotted against an increase in temperature from 15 to 95 °C. The single melt peak indicates that RNA is being released, and the corresponding temperature was denoted as T_m_.

To determine the thermal stability of the FMDVs, we performed a particle stability thermal release assay (PaSTRy) using a nucleic intercalating dye to detect exposure of the viral genome. The fundamental principle of PaSTRy involves applying a temperature gradient from room temperature to high temperature to purified virus particles. As the temperature increases, virus particles undergo structural changes leading to dissociation, thereby releasing the virus genome. Eight different strains of FMDV serotypes O and A were purified by SDG centrifugation and subjected to PaSTRy. Even within the same serotype, distinct differences in melting temperature (T_m_) values were observed, indicating variations in thermal stability. Notably, serotype O viruses exhibited relatively lower stability compared to serotype A. Among various strains of FMDV, the domestic isolate A Yeoncheon virus demonstrated the highest stability with a T_m_ of 54 °C. In comparison, the O Jincheon virus showed notable instability with a T_m_ of 43 °C (Figure 1).

### 3.2. Effect of Improving Thermal Stability of FMDV by Calcium Treatment

We evaluated the effect of improving thermal stability by adding calcium chloride as a stabilizer. Purified FMDVs were treated with different concentrations of calcium chloride and their effect on thermal stability was analyzed using PaSTRy. All FMDV strains were found to have improved thermal stability after calcium treatment, and the stabilization effect of the 146S particles increased in a dose-dependent manner (Table 1). However, treatment with concentrations exceeding 30 mM exhibited a gradual destabilization trend of 146S particles, and at 50 mM, viral stability was significantly reduced compared to the calcium-untreated control. The optimal calcium concentration that achieved the highest stabilizing effect among all the FMDV strains was 10 mM.

### 3.3. Evaluation of FMDV Stability for Long-Term Storage

**Figure 2:**
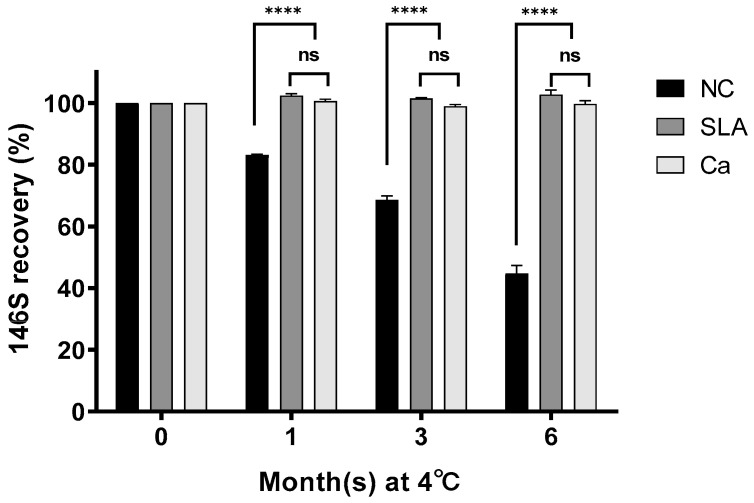
Effect of excipients for cold chain storage of FMDV. Unformulated, SLA, and calcium-formulated FMDV O Jincheon 146S antigens were stored at 4 °C for 6 months. The amount of 146S particles was measured by high-performance liquid chromatography, and the recovery rate was expressed as a percentage (%). ****, *p* < 0.0001; ns, not significant. Abbreviations: NC, negative control (no excipients); SLA, 10% sucrose with 5% lactalbumin hydrolysate; Ca, 10 mM calcium chloride.

We evaluated the potential of calcium chloride for the long-term storage of FMD vaccine antigens. SLA, a potent stabilizer, was used as a positive control. The purified O Jincheon virus was added to 10 mM calcium chloride and stored at 4 °C for 6 months (Figure 2). Recovery rates of the 146S antigen were measured at one, three, and six months. In the untreated group (NC), the 146S recovery rate decreased over time. Specifically, 80% of the 146S antigen was recovered at one month, decreased to 68% at three months, and further declined to 45% at six months. In contrast, the calcium-treated group maintained 146S antigen integrity throughout the storage period, with no significant antigen loss observed. Additionally, the stabilizing effect of calcium chloride was significantly higher (*p* = 0.0001) than in the untreated group and was equivalent to SLA, a potent stabilizer.

### 3.4. Immunogenicity of FMD Vaccines Containing Stabilizers in Pigs

**Figure 3:**
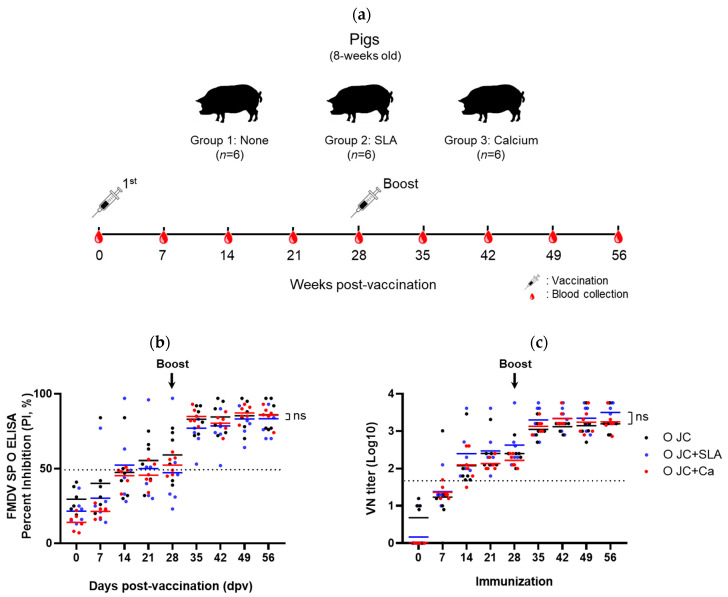
Immunogenicity of FMD vaccines containing stabilizing excipients. Antibodies elicited by immunization with FMD O Jincheon vaccines prepared by adding SLA (10% sucrose with 5% lactalbumin hydrolysate) and calcium (10 mM). (**a**) Schematic representation of the animal experiment. Six pigs per group were vaccinated twice at 4-week intervals, and blood samples were collected weekly. (**b**) Enzyme-linked immunosorbent assay (ELISA) was used to measure the antibody titers of the structural protein (SP). The antibody values were expressed as percent inhibition (PI) values. A PI value of ≥50% (dotted line) was considered to indicate a positive result. (**c**) The homologous virus-neutralizing (VN) antibody titers were measured using the virus neutralization test. A titer ≥1:45 (1.65 log_10_) (dotted line) was considered to indicate a positive result. Abbreviations: O JC, O Jincheon virus; SLA, 10% sucrose with 5% lactalbumin hydrolysate; Ca, 10 mM calcium chloride; ns, not significant.

The immunogenicity of the FMD vaccines formulated with the addition of stabilizers, calcium chloride, and SLA, was evaluated. Six pigs per group (group 1: none, group 2: SLA, and group 3: calcium chloride), aged 8 weeks, were vaccinated twice via intramuscular injection at 4-week intervals, and blood samples were collected weekly until 56 dpv (Figure 3a). SP- and virus-neutralizing (VN) antibody levels consistently increased in all groups until 56 dpv. For SP antibodies, the percent inhibition (PI) value was confirmed positive (≥50%) in all pigs starting from 35 dpv, and there was no significant difference between groups (O Jincheon vs. SLA, *p* = 0.4749; O Jincheon vs. calcium, *p* = 0.3075; SLA vs. calcium, *p* = 0.0935) (Figure 3b). The VN antibody titers against the homologous virus (O/Jincheon/SKR/2014) were above 1:45 (1.65 log_10_) for all pigs after 21 dpv, and the subsequent titers were approximately 1:1000 (3.00 log_10_) with the booster vaccination. The titers of the calcium chloride group were equivalent to those of the other groups (O Jincheon vs. SLA, *p* = 0.6672; O Jincheon vs. calcium, *p* = 0.8816; SLA vs. calcium, *p* = 0.7779) (Figure 3c). This indicates that calcium chloride did not have a negative effect on the immunogenicity of the FMD vaccine 146S antigen in pigs.

## 4. Discussion

Vaccination policy has been implemented nationwide for cattle, pigs, and goats since a large-scale outbreak of FMD occurred between November 2010 and April 2011, causing approximately KRW 3 trillion in economic damage in South Korea. The three types of FMD vaccines currently in use are imported from foreign countries; therefore, we are planning to build a domestic FMD vaccine manufacturing facility and have developed seed viruses for the FMD vaccine.

While evaluating the seed viruses, we found that the O Jincheon virus is physically unstable. To address this instability, we developed a stabilizing agent composed of SLA [13]. However, the sticky characteristics of SLA make it difficult to mix with vaccine excipients and pose a risk of microbial contamination. In this study, we sought a simple alternative to SLA as a vaccine stabilizer and found that calcium enhanced the stability of the physically unstable type O Jincheon.

In the production of many viral vaccines, the antigenic content is sometimes calculated from the amount of a subunit protein; however, in the case of the FMD vaccine, the content of the intact viral particle (146S) must be measured, not the subunit protein, because degradation of the 146S particle to the pentamer (12S) significantly reduces the immunogenicity of the vaccine [15]. Therefore, the antigen content of the FMD vaccine was measured by purifying the viral particles via ultracentrifugation [39,44] or by HPLC fitted with a gel column that separates the material by particle size [37]. Ultimately, the amount of 146S particles recovered is the linchpin for FMD vaccine production. Therefore, it is essential to develop stabilizers to overcome the instability of the antigen, as FMDVs may be destroyed during the process of manufacturing the FMD vaccine antigen, reducing its value as a vaccine antigen.

Previous studies investigating the thermal stability of FMDV serotypes showed that SAT serotype viruses were the least thermostable, followed by the O, C, Asia1, and A serotypes [11]. Therefore, many researchers have conducted studies to improve the stability of FMDV, mainly targeting SAT serotypes, and the PaSTRy method has been widely used as a method to evaluate the stability of FMDVs [22,27,45,46,47].

In this study, we applied the PaSTRy method to our in-house-developed FMD type O and A viruses and found that the type O Jincheon virus showed the lowest Tm value, indicating that it was highly unstable. It was previously reported that the type O Jincheon virus is much more easily degraded at 45 °C compared to other FMDVs [35], and the PaSTRy method in the current study once again demonstrated the instability of the type O Jincheon virus.

Divalent transition metal ions, including calcium, contribute to stabilization by forming salt bridges at the inter-pentameric interface of the FMDV capsid, and this effect is known to be viral-strain-dependent [27]. We tested the stability of domestic isolates by treating them with calcium, nickel, and copper and found that nickel and copper had no effect, whereas calcium improved the stability of both type O and type A isolates. The result measured by the PaSTRy method exhibited an increasing trend in T_m_ values up to 10 mM calcium and a decreasing trend in T_m_ values above 30 mM calcium, which is consistent with previous reports that treatment with 0.5 M calcium resulted in lower T_m_ values than the untreated control [27,46]. Our results suggest that FMDV stability may be reduced by high concentrations of calcium ions. In addition, other researchers reported that the stabilizing effect of calcium was similar to ours, with the highest T_m_ values in the combination of 10 mM calcium and 0.2–0.4 M NaCl [22].

Given that FMD vaccines are usually stored under refrigerated conditions, it is important to evaluate their stability under these conditions. Therefore, we aimed to evaluate the stabilizing effect of calcium chloride on the unstable strain type O Jincheon virus. As expected, the vaccine caused a significant decrease in the amount of antigens over time. In contrast, the addition of calcium enhanced the preservation stability, equivalent to that of SLA, an earlier stabilizer. In a previous study evaluating the stability of FMDVs at different temperatures, FMDVs originating from China showed little change in the amount of antigen at 4 °C, while the amount of antigen decreased by approximately 90% in 6 h at 37 °C [17]. FMDVs isolated in Argentina decreased in the amount of antigen by approximately 90% in 24 h at 37 °C [34]. While many FMDVs are thermally unstable at 37 °C, it is unusual for an FMDV to be so unstable at 4 °C, as was the case (type O Jincheon virus) in this study. We have previously reported that the type O Jincheon virus is unstable, resulting in a wide spectrum of stability depending on the type of buffer solution, indicating its inherently low stability [35]. Although the enhanced stability of type O Jincheon by calcium chloride has been confirmed, it is necessary to investigate the immunogenicity of the antigen supplemented with calcium chloride in target animals to ultimately evaluate its utilization as a vaccine. The efficacy of the FMD vaccine is determined by the viral particle called 146S. It is already known that if the intact 146S of the FMDV is degraded into the pentameric 12S, the efficacy of the FMD vaccine is significantly reduced [15]. Therefore, it was not necessary to evaluate the immunogenicity of samples with and without 12S in pigs (Figure 2). In this study, we aimed to evaluate whether the addition of calcium that increased the stability of the type O Jincheon virus could affect its immunogenicity in pigs. In addition, since the experimental vaccine was prepared by purifying and quantifying only 146S particles, the degraded form, 12S, was not included in the experimental vaccine.

The antibody titers were equivalent to those of the control antigens, confirming their potential as FMD vaccines. In addition, the VN titer reached approximately 1:1000 post-booster, suggesting that the vaccine is predicted to protect against FMDVs, based on the correlation between VN titers and in vivo protection [48,49].

Various strategies have been reported to enhance the stability of FMDVs. These include substituting the amino acid residues involved in electrostatic repulsion [50,51,52] or strengthening the interactions between pentamers [53,54]. However, SLA was developed as an easier way to improve FMDV stability than to create FMDV variants, and calcium chloride was discovered as a novel stabilizing agent to overcome the handling difficulties of SLA. As shown in this study, the immunogenicity of the SLA group and the calcium chloride group was equivalent in pigs. Therefore, the use of calcium chloride as a vaccine stabilizer, which is easier to mix and store with various vaccine excipients than SLA, could be widely useful for not only FMD vaccines but also for other veterinary vaccines.

## 5. Conclusions

FMDV O Jincheon has been difficult to use as an FMD vaccine because it is easily dissociated during storage. However, by treating the FMDV O Jincheon with calcium chloride, it can be employed as a vaccine candidate. These findings are expected to contribute to the development of stable vaccine formulation, especially for inherently unstable FMDV strains.

## Figures and Tables

**Table 1:** Thermal stability of FMDVs according to calcium concentration by PaSTRy.

FMDV		Calcium Chloride Concentration (mM)				
		0	3	10	30	50
		Melt Peak (T_m_, °C)				
**O**	**Boeun**	52.0	53.0	54.0	50.0	45.5
**O**	**Andong**	47.5	50.5	53.5	52.0	45.5
**O**	**Jincheon**	43.0	47.0	51.0	46.0	43.0
**O**	**PanAsia2**	49.0	51.0	54.5	48.0	44.0
**O**	**Taiwan**	49.5	50.5	52.0	NA	NA
**A**	**Yeoncheon**	54.0	54.5	55.5	53.0	45.0
**A**	**Pocheon**	49.0	51.0	52.5	48.0	44.5
**A_22_**	**Iraq**	53.5	52.5	54.5	54.0	NA

The value of ≥ 50 °C was considered stable, and the change in color from green to yellow shows a decrease in the stability of the FMDV capsid (146S). NA, not available.

## Data Availability

Data are contained within the article.
